# Beyond Glycemic Control: Real-World 12-Month Effects of Insulin Glargine/Lixisenatide on Weight, Endogenous Insulin Secretion, and Albuminuria

**DOI:** 10.3390/jcm15135049

**Published:** 2026-06-29

**Authors:** Sadettin Ozturk, Elif Melis Baloğlu Akyol

**Affiliations:** Department of Endocrinology and Metabolism, Gaziantep City Hospital, 27100 Gaziantep, Turkey; melis_baloglu@hotmail.com

**Keywords:** type 2 diabetes mellitus, iGlarLixi, insulin glargine, lixisenatide, HbA1c, albuminuria, C-peptide, real-world study, endogenous insulin secretion

## Abstract

**Background:** Fixed-ratio combinations of basal insulin and glucagon-like peptide-1 receptor agonists have emerged as an effective strategy for treatment intensification in type 2 diabetes mellitus (T2DM). However, long-term real-world data on their metabolic, β-cell, and renal effects remain limited. This study aimed to evaluate the 12-month real-world outcomes of insulin glargine/lixisenatide (iGlarLixi) therapy. **Methods:** This retrospective observational study included 78 patients with T2DM who were initiated on iGlarLixi and followed for 12 months. Clinical and laboratory parameters were assessed at baseline, 3 months, and 12 months. Changes in anthropometric, glycemic, biochemical, and renal parameters were analyzed. Early (3-month) changes were evaluated as predictors of long-term outcomes using correlation and receiver operating characteristic (ROC) analyses. **Results:** Median HbA1c decreased from 9.0% to 8.2% at 12 months (*p* = 0.015), with clinically meaningful improvement (≥1% reduction) observed in 21 (26.9%) patients. Body weight decreased significantly from 93.0 kg to 89.5 kg (*p* < 0.001). C-peptide levels increased from 2.46 to 3.00 ng/mL (*p* = 0.016). Median albumin-to-creatinine ratio (ACR) showed statistically significant paired changes; however, group-level median values remained similar over time. A reduction in albuminuria was observed in 34 (54.8%) of patients with available paired data. Exploratory ROC analyses suggested that early changes in body weight (AUC: 0.82), HbA1c (AUC: 0.74), and C-peptide (AUC: 0.76) may be associated with long-term outcomes. A combined model incorporating early weight and C-peptide changes showed improved exploratory predictive performance (AUC: 0.88). **Conclusions:** In a real-world setting, iGlarLixi therapy was associated with significant improvements in glycemic control and body weight, along with increased C-peptide levels that may reflect improved endogenous insulin secretion. While group-level renal changes were modest, individual-level improvements in albuminuria were observed in a substantial proportion of patients. Early treatment responses may serve as practical predictors of long-term outcomes, supporting a personalized approach to diabetes management. These findings should be interpreted in the context of concomitant therapies and comorbidities that may have influenced metabolic and renal outcomes in routine clinical practice.

## 1. Introduction

Type 2 diabetes mellitus (T2DM) is a progressive metabolic disorder characterized by chronic hyperglycemia, insulin resistance, progressive β-cell dysfunction, and a high burden of cardiovascular and renal complications [1,2]. Despite the availability of multiple glucose-lowering therapies, many patients fail to achieve durable glycemic control, particularly as disease duration increases and endogenous insulin secretory capacity declines. In clinical practice, treatment intensification is often required when oral antidiabetic agents and/or basal insulin are insufficient to maintain individualized glycemic targets. However, intensification with insulin alone may be limited by weight gain, hypoglycemia, treatment complexity, and poor adherence, all of which can negatively affect long-term outcomes [3,4].

Current treatment strategies increasingly emphasize a patient-centered approach that targets not only HbA1c reduction but also body weight, hypoglycemia risk, cardiovascular risk, renal protection, treatment burden, and long-term metabolic durability [5,6]. The 2026 American Diabetes Association Standards of Care continue to support individualized pharmacologic therapy in T2DM and highlight the importance of agents with broader cardiometabolic benefits, including glucagon-like peptide-1 receptor agonists (GLP-1 RAs), especially in patients requiring treatment intensification beyond oral therapy or basal insulin [7,8,9,10]. In this context, fixed-ratio combinations of basal insulin and GLP-1 RA have emerged as an attractive therapeutic option because they combine complementary mechanisms of action in a single injectable formulation.

Insulin glargine/lixisenatide fixed-ratio combination (iGlarLixi; Soliqua) combines insulin glargine 100 U/mL, which primarily targets fasting plasma glucose through sustained basal insulin replacement, with lixisenatide, a short-acting GLP-1 RA that improves postprandial glucose control by enhancing glucose-dependent insulin secretion, suppressing glucagon secretion, slowing gastric emptying, and promoting satiety [11,12]. This dual mechanism may provide effective glycemic control while mitigating some disadvantages of basal insulin intensification, particularly insulin-associated weight gain and hypoglycemia. Compared with basal insulin alone, iGlarLixi has demonstrated greater HbA1c reduction, higher rates of achieving HbA1c targets, and favorable effects on body weight in randomized controlled trials [13,14,15].

The LixiLan clinical trial program established the efficacy and safety of iGlarLixi in patients with T2DM inadequately controlled on oral antidiabetic drugs or basal insulin. Compared with insulin glargine alone, iGlarLixi provided greater HbA1c reduction while limiting weight gain and maintaining an acceptable hypoglycemia profile. These benefits were consistently observed across different patient subgroups, supporting the use of iGlarLixi as an effective treatment intensification strategy in routine clinical practice [16,17,18,19,20,21].

Although randomized controlled trials provide high-quality evidence, real-world data are essential to understand treatment effectiveness under routine clinical conditions, where patients are more heterogeneous and adherence, comorbidities, dose titration, and follow-up intensity may differ substantially from trial settings. Real-world studies have generally confirmed that iGlarLixi improves glycemic control with neutral or favorable effects on body weight and a low risk of hypoglycemia [22,23,24]. However, most available real-world studies have relatively short follow-up periods, commonly around 24–30 weeks, and longer-term data remain limited.

Beyond glycemic control and body weight, the potential renal and β-cell-related effects of GLP-1 RA-based therapies are increasingly important. Diabetic kidney disease remains one of the most common and clinically significant complications of T2DM, and albuminuria is a key early marker of renal microvascular injury and future cardiovascular risk. GLP-1 RAs have been associated with reductions in albuminuria and slower progression of diabetic kidney disease in several clinical and mechanistic studies, although the magnitude and mechanisms of these effects may vary between agents and populations [25,26,27,28]. Proposed mechanisms include improved glycemic control, weight reduction, blood pressure lowering, anti-inflammatory effects, natriuresis, and direct renal actions mediated through GLP-1 receptor pathways [29,30]. Recent mechanistic reviews have further highlighted the complex and potentially heterogeneous renal effects of GLP-1 receptor agonists, suggesting that renal benefits may vary according to individual pharmacologic properties and target pathways [31]. Lixisenatide has also been investigated in relation to renal outcomes, with evidence suggesting possible beneficial effects on albuminuria and renal parameters in patients with T2DM [32,33,34]. Unlike longer-acting GLP-1 receptor agonists, lixisenatide is a short-acting agent with a predominant effect on postprandial glucose control through delayed gastric emptying. In addition, experimental and clinical studies suggest that renal effects of GLP-1 receptor agonists may not be entirely class-uniform and could vary according to pharmacokinetic and pharmacodynamic properties. Therefore, evaluation of lixisenatide-containing fixed-ratio combinations may provide clinically relevant information beyond class-level observations. These characteristics may be particularly relevant when interpreting outcomes related to postprandial glucose control, endogenous insulin secretion, and albuminuria, which were key endpoints of the present study.

C-peptide is a clinically useful marker of endogenous insulin secretion and residual β-cell function. In patients with T2DM, preservation or improvement of C-peptide levels may reflect improved β-cell responsiveness, reduced glucotoxicity, or enhanced metabolic stability [35]. Because GLP-1 RAs stimulate insulin secretion in a glucose-dependent manner and may reduce β-cell stress through improved glycemic and weight control, evaluation of C-peptide changes during iGlarLixi therapy may provide additional insight beyond HbA1c alone [36,37,38]. However, real-world studies examining the relationship between iGlarLixi treatment, C-peptide dynamics, weight change, and renal markers such as albumin-to-creatinine ratio remain scarce.

The primary objective of this study was to evaluate the 12-month effects of iGlarLixi therapy on glycemic control, body weight, and renal parameters in patients with T2DM treated in routine clinical practice. Secondary objectives included assessment of longitudinal changes in C-peptide levels as a marker of endogenous insulin secretion. Exploratory analyses were performed to investigate associations between early treatment responses and long-term clinical outcomes.

## 2. Materials and Methods

### 2.1. Study Design and Population

The study protocol was reviewed and approved by the Gaziantep City Hospital Non-Interventional Clinical Research Ethics Committee (Decision No: 458/2026, Date: 6 March 2026). The study was conducted in accordance with the principles of the Declaration of Helsinki. As this was a retrospective study using anonymized data, informed consent requirements were handled in accordance with institutional regulations.

**Figure 1:**
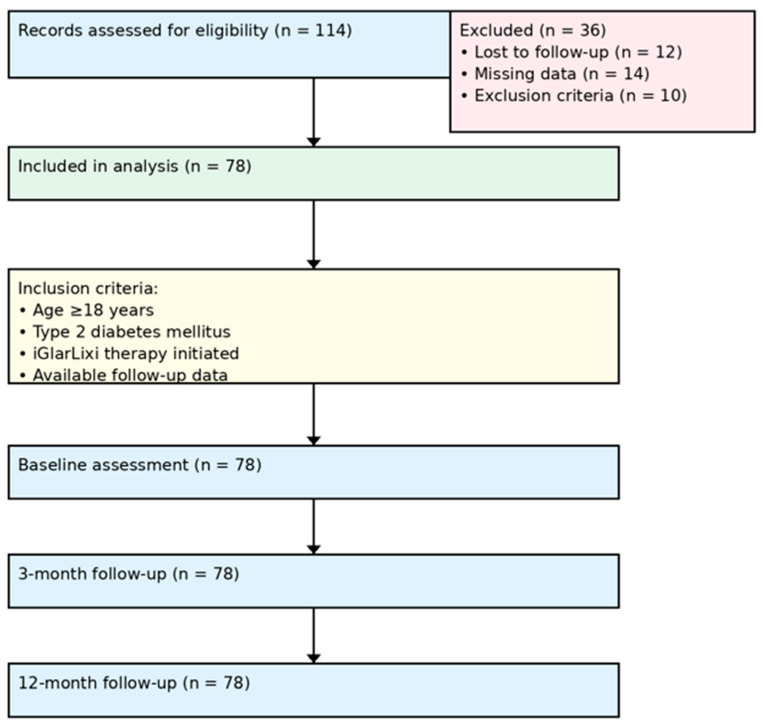
Flowchart of the Study.

This retrospective observational study was conducted at the Department of Endocrinology and Metabolism of Gaziantep City Hospital. The study included adult patients with type 2 diabetes mellitus (T2DM) who were initiated on a fixed-ratio combination therapy of insulin glargine and lixisenatide (iGlarLixi; Soliqua) and followed for up to 12 months under routine clinical practice conditions. Patients were identified through the hospital’s electronic medical records system. Clinical and laboratory data at baseline (prior to treatment initiation), 3 months, and 12 months were retrospectively collected and analyzed. Patients were excluded if they had incomplete follow-up data, missing laboratory measurements required for longitudinal analyses, discontinued follow-up before 12 months, or met predefined exclusion criteria. Specifically, among 114 eligible patients, 36 were excluded due to loss to follow-up (n = 12), missing data (n = 14), or other exclusion criteria (n = 10), as illustrated in Figure 1.

### 2.2. Data Collection and Variables

Clinical, demographic, and laboratory data were retrospectively extracted from the electronic medical records of Gaziantep City Hospital. Data were collected at three predefined time points: baseline (prior to initiation of insulin glargine/lixisenatide therapy), 3 months, and 12 months of follow-up. Baseline was defined as the closest available clinical and laboratory assessment performed before the initiation of insulin glargine/lixisenatide therapy. For each patient, baseline values were selected from routine outpatient records obtained within 30 days prior to iGlarLixi initiation. If more than one measurement was available during this period, the value closest to the treatment initiation date was used. Measurements obtained after treatment initiation were not considered baseline values. Demographic and clinical variables included age, sex, duration of diabetes, body weight, body mass index (BMI), and treatment dose of insulin glargine/lixisenatide. Glycemic parameters comprised fasting plasma glucose and glycated hemoglobin (HbA1c). Renal function was assessed using serum urea, creatinine, estimated glomerular filtration rate (eGFR), spot urine albumin-to-creatinine ratio (ACR), and protein-to-creatinine ratio. Hepatobiliary evaluation included liver enzymes (ALT, AST, GGT, ALP) and ultrasonographic assessment of hepatic steatosis. Metabolic and hematological variables included lipid profile (total cholesterol, LDL-C, HDL-C, triglycerides), complete blood count parameters, vitamin D, vitamin B12, and C-peptide levels as a marker of endogenous insulin secretion. To evaluate temporal changes, delta (Δ) values were calculated for both 3-month (Δ3) and 12-month (Δ12) intervals, and these were used in correlation analyses to explore relationships between early and long-term treatment responses.

Information regarding major comorbidities and concomitant medications at the time of iGlarLixi initiation was also collected from electronic medical records. Recorded comorbidities included hypertension, dyslipidemia, coronary artery disease, obesity, and chronic kidney disease. Concomitant antidiabetic therapies included metformin, sodium-glucose cotransporter-2 inhibitors (SGLT2 inhibitors), dipeptidyl peptidase-4 inhibitors (DPP-4 inhibitors), sulfonylureas, and basal insulin. The use of renin–angiotensin system blockers (ACE inhibitors or angiotensin receptor blockers) and statins was also recorded because of their potential influence on renal and metabolic outcomes.

As this study reflects routine clinical practice, insulin dose titration was performed at the discretion of the treating endocrinologist according to individual glycemic targets and tolerability. Formal adherence assessments were not routinely available in the electronic medical records. Standard dietary and lifestyle recommendations were provided as part of routine diabetes care; however, adherence to these interventions was not systematically recorded and therefore could not be analyzed. Information regarding baseline concomitant medications was available; however, longitudinal changes in concomitant therapies, including treatment initiation, discontinuation, or dose adjustments during follow-up, were not systematically recorded in the electronic medical records and therefore could not be evaluated.

### 2.3. Outcome Measures

The primary outcome of this study was the change in glycemic control over 12 months, as assessed by HbA1c levels. Secondary outcomes included changes in body weight and BMI, reflecting the metabolic impact of insulin glargine/lixisenatide therapy.

Secondary outcomes comprised longitudinal changes in renal parameters, including serum creatinine, estimated glomerular filtration rate (eGFR), spot urine albumin-to-creatinine ratio (ACR), and protein-to-creatinine ratio, to evaluate potential renal effects of treatment. In addition, changes in C-peptide levels were assessed as a surrogate marker of endogenous insulin secretion.

Exploratory outcomes included changes in lipid profile parameters (total cholesterol, LDL-C, HDL-C, triglycerides), liver enzymes, and other metabolic biomarkers. Furthermore, temporal relationships between early (3-month) and long-term (12-month) treatment responses were analyzed using calculated delta (Δ) values. Correlation analyses were performed to investigate associations between weight change, glycemic improvement, C-peptide dynamics, treatment dose, and renal parameters, particularly albuminuria.

### 2.4. Statistical Analysis

All statistical analyses were performed using IBM SPSS Statistics version 27.0 (IBM Corp., Armonk, NY, USA) and R software (version 4.3.0; R Foundation for Statistical Computing, Vienna, Austria). Continuous variables were expressed as median and interquartile range (IQR), while categorical variables were presented as frequencies and percentages. The normality of data distribution was assessed using the Shapiro–Wilk test. As most variables did not follow a normal distribution, non-parametric methods were applied. Comparisons of repeated measurements across three time points (baseline, 3 months, and 12 months) were performed using the Friedman test. When significant differences were detected, pairwise comparisons were conducted using the Wilcoxon signed-rank test with appropriate adjustment for multiple comparisons. Changes over time were further quantified by calculating delta (Δ) values for 3-month (Δ3) and 12-month (Δ12) intervals. Correlations between these changes were evaluated using Spearman’s rank correlation coefficient. The strength of correlations was interpreted as weak (r < 0.3), moderate (0.3–0.5), or strong (>0.5). Receiver operating characteristic (ROC) analyses were performed as exploratory analyses to investigate whether early treatment-related changes could identify patients more likely to achieve predefined long-term outcomes. Given the relatively small sample size, these analyses were intended for hypothesis generation rather than definitive prediction modeling. Net Reclassification Improvement (NRI) and Decision Curve Analysis (DCA) were additionally performed as exploratory assessments of potential incremental predictive value, and their results should be interpreted cautiously. Event rates for predefined outcomes were calculated according to clinically meaningful thresholds, including ≥3 kg weight loss, ≥1% HbA1c reduction, and albuminuria reduction at 12 months. A two-tailed *p*-value < 0.05 was considered statistically significant.

A post hoc power analysis was performed to assess the statistical adequacy of the study sample. Based on the observed change in HbA1c levels between baseline and 12 months (effect size estimated from paired differences), the calculated effect size was moderate (Cohen’s d ≈ 0.5). With a final sample size of 78 patients who completed the 12-month follow-up, a significance level (α) of 0.05, and a two-tailed hypothesis, the statistical power (1 − β) was estimated to be approximately 0.80 (80%), indicating adequate power to detect clinically meaningful differences in primary outcomes. Power calculations were performed using G*Power software (version 3.1; Heinrich Heine University, Düsseldorf, Germany).

## 3. Results

A total of 114 patients with type 2 diabetes mellitus who initiated insulin glargine/lixisenatide therapy were assessed for eligibility. Of these, 36 patients were excluded, and 78 patients completed the 12-month follow-up and were included in the final analysis. The study population consisted of 61 women (78.2%) and 17 men (21.8%). The median duration of diabetes was 10 years (IQR: 8). At baseline, the median body weight was 93.0 kg (IQR: 17.98), and the median body mass index was 35.65 kg/m^2^ (IQR: 7.11). The median fasting plasma glucose level was 182.0 mg/dL (IQR: 113.0), and the median HbA1c level was 9.0% (IQR: 2.30). The median serum creatinine was 0.725 mg/dL (IQR: 0.31), and the median eGFR was 95.0 mL/min/1.73 m^2^ (IQR: 31.0). The median albumin-to-creatinine ratio was 15.30 mg/g (IQR: 44.03), and the median protein-to-creatinine ratio was 88.5 mg/g (IQR: 81.65). The median C-peptide level was 2.46 ng/mL (IQR: 2.49) (Table 1).

At baseline, hypertension and dyslipidemia were the most common comorbidities, affecting 61.5% and 66.7% of patients, respectively. Obesity (BMI ≥ 30 kg/m^2^) was present in 78.2% of the cohort. At baseline, 33 patients (42.3%) had elevated albuminuria (ACR ≥ 30 mg/g), including 26 patients with microalbuminuria and 7 patients with macroalbuminuria. Regarding concomitant therapies, metformin was the most frequently prescribed glucose-lowering medication (85.9%), followed by SGLT2 inhibitors (48.7%) and DPP-4 inhibitors (30.8%). More than half of the patients were receiving renin–angiotensin system blockers (57.7%) and statins (62.8%) at the time of iGlarLixi initiation (Table 2).

Changes in anthropometric and glycemic parameters over time are presented in Table 3. Median body weight decreased from 93.0 kg (IQR: 18.25) at baseline to 91.0 kg (IQR: 17.0) at 3 months and 89.5 kg (IQR: 17.0) at 12 months, with statistically significant differences across all time intervals (all *p* < 0.001), corresponding to a large effect size (r = 0.52). Median BMI decreased from 35.65 kg/m^2^ (IQR: 7.32) to 35.41 kg/m^2^ (IQR: 7.18) at 3 months and 34.74 kg/m^2^ (IQR: 7.11) at 12 months (all *p* < 0.001; r = 0.50), indicating a moderate-to-large effect.

Median fasting plasma glucose decreased from 182.0 mg/dL (IQR: 113.0) at baseline to 168.0 mg/dL (IQR: 95.0) at 3 months and 165.0 mg/dL (IQR: 100.0) at 12 months; however, these changes were not statistically significant (*p* = 0.612 for 0–3 months, *p* = 0.085 for 0–12 months, and *p* = 0.210 for 3–12 months; r = 0.18). Median HbA1c remained unchanged at 3 months (9.0% vs. 9.0%) but decreased to 8.2% (IQR: 2.33) at 12 months. Clinically meaningful glycemic improvement, defined as a ≥1% reduction in HbA1c, was observed in 21 (26.9%) of patients at 12 months. This reduction was statistically significant between baseline and 12 months (*p* = 0.015) and between 3 and 12 months (*p* = 0.006), with a moderate effect size (r = 0.34). No significant change was observed between baseline and 3 months (*p* = 0.521) (Table 3).

Changes in renal function parameters over time are presented in Table 4. Median serum creatinine levels remained largely stable over the study period, with a small but statistically significant difference observed between baseline and 3 months (*p* = 0.022), although this change was not sustained at 12 months (*p* = 0.776), indicating a negligible effect size (r = 0.12). No significant changes were observed in eGFR across time points (all *p* > 0.05; r = 0.10). The median albumin-to-creatinine ratio (ACR) showed a statistically significant paired change from baseline to 12 months (*p* = 0.012) and between 3 and 12 months (*p* = 0.006), with a small effect size (r = 0.28). However, median ACR values at baseline (15.30 mg/g) and 12 months (15.34 mg/g) were comparable, indicating that the observed statistical significance reflects variability in individual-level changes rather than a consistent group-level reduction. A reduction in albuminuria, defined as a decrease in ACR from baseline to 12 months, was observed in 34 (54.8%) of patients with available paired data. In contrast, the protein-to-creatinine ratio demonstrated a consistent and statistically significant decrease over time (*p* = 0.005 for 0–3 months, *p* = 0.002 for 0–12 months, and *p* = 0.020 for 3–12 months; r = 0.32), corresponding to a moderate effect size (Table 4).

Changes in lipid profile and biochemical parameters are shown in Table 5. Median total cholesterol levels were 209 mg/dL (IQR: 52) at baseline, 202 mg/dL (IQR: 58) at 3 months, and 194 mg/dL (IQR: 43.5) at 12 months (*p* = 0.764 for 0–3 months, *p* = 0.002 for 0–12 months, and *p* = 0.022 for 3–12 months; r = 0.30). Median LDL cholesterol levels were 113.5 mg/dL (IQR: 47.5) at baseline, 111 mg/dL (IQR: 29) at 3 months, and 102.86 mg/dL (IQR: 45) at 12 months (*p* = 0.748 for 0–3 months, *p* = 0.096 for 0–12 months, and *p* = 0.016 for 3–12 months; r = 0.26). Median HDL cholesterol levels were 52 mg/dL (IQR: 15) at baseline, 52 mg/dL (IQR: 15) at 3 months, and 50 mg/dL (IQR: 15.6) at 12 months (*p* = 0.512 for 0–3 months, *p* = 0.399 for 0–12 months, and *p* = 0.283 for 3–12 months; r = 0.10). Median triglyceride levels were 201 mg/dL (IQR: 117) at baseline, 193 mg/dL (IQR: 120.9) at 3 months, and 196 mg/dL (IQR: 112.5) at 12 months (*p* = 0.876 for 0–3 months, *p* = 0.381 for 0–12 months, and *p* = 0.686 for 3–12 months; r = 0.08). Median C-peptide levels were 2.46 ng/mL (IQR: 2.59) at baseline, 2.66 ng/mL (IQR: 2.53) at 3 months, and 3.00 ng/mL (IQR: 2.93) at 12 months (*p* = 0.030 for 0–3 months, *p* = 0.016 for 0–12 months, and *p* = 0.001 for 3–12 months; r = 0.33). Median TSH levels were 1.96 mIU/L (IQR: 1.39) at baseline, 1.69 mIU/L (IQR: 1.18) at 3 months, and 1.76 mIU/L (IQR: 1.04) at 12 months (*p* = 0.081 for 0–3 months, *p* = 0.009 for 0–12 months, and *p* = 0.264 for 3–12 months; r = 0.24). Median uric acid levels were 4.61 mg/dL (IQR: 2.01) at baseline, 4.71 mg/dL (IQR: 1.72) at 3 months, and 4.75 mg/dL (IQR: 1.89) at 12 months (*p* = 0.386 for 0–3 months, *p* = 0.289 for 0–12 months, and *p* = 0.280 for 3–12 months; r = 0.09) (Table 5).

**Figure 2:**
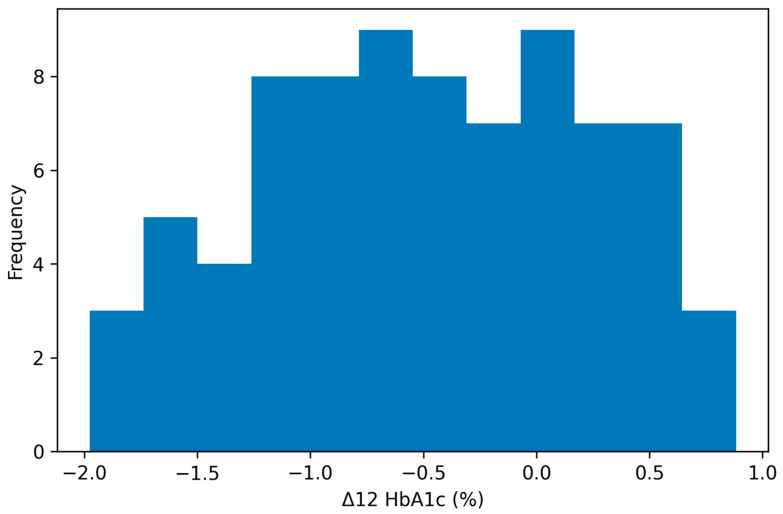
Distribution of individual 12-month HbA1c changes.

The apparent discrepancy between group-level median HbA1c values and median paired ΔHbA1c values reflects the use of paired individual-level calculations. Therefore, median Δ values should not be interpreted as the arithmetic difference between the corresponding group medians. To further clarify this issue, the distribution of individual Δ12 HbA1c values is presented in Figure 2. This figure demonstrates substantial inter-individual variability in HbA1c response and supports the interpretation that paired individual-level Δ values may differ from the arithmetic difference between group-level median values (Figure 2).

Median changes (Δ3 and Δ12) in key clinical parameters are shown in Table 6. Median body weight change was −1.88 kg (IQR: 1.00) at 3 months and −3.13 kg (IQR: 2.55) at 12 months (*p* < 0.001; r = 0.52). Median HbA1c change was −0.03% (IQR: 1.95) at 3 months and −0.20% (IQR: 2.35) at 12 months (*p* = 0.015; r = 0.34). Median C-peptide change was +0.27 ng/mL (IQR: 1.31) at 3 months and +0.51 ng/mL (IQR: 1.67) at 12 months (*p* = 0.016; r = 0.33). Median albumin-to-creatinine ratio change was −1.85 mg/g (IQR: 18.60) at 3 months and −2.56 mg/g (IQR: 19.85) at 12 months (*p* = 0.012; r = 0.28) (Table 6).

Correlation analysis between Δ3 and Δ12 changes is shown in Table 7. A strong positive correlation was observed between Δ3 and Δ12 body weight changes (r = 0.801, *p* < 0.001). Δ3 and Δ12 HbA1c changes were strongly correlated (r = 0.639, *p* < 0.001), as were Δ3 and Δ12 C-peptide changes (r = 0.658, *p* < 0.001). A weak negative correlation was found between Δ3 weight and Δ12 HbA1c (r = −0.268, *p* = 0.018). Δ3 weight showed a moderate negative correlation with Δ3 C-peptide (r = −0.370, *p* = 0.001), while Δ12 weight demonstrated a weak negative correlation with Δ12 C-peptide (r = −0.262, *p* = 0.021). Δ12 C-peptide was moderately negatively correlated with Δ3 albumin-to-creatinine ratio (r = −0.309, *p* = 0.006) and weakly negatively correlated with Δ12 albumin-to-creatinine ratio (r = −0.243, *p* = 0.035). No significant correlation was observed between Δ3 HbA1c and Δ3 weight (r = −0.092, *p* = 0.425), and the correlation between Δ12 HbA1c and Δ12 weight was not statistically significant (r = −0.221, *p* = 0.054) (Table 7).

Associations between treatment dose and clinical outcomes are shown in Table 8. Baseline iGlarLixi dose was positively correlated with Δ3 HbA1c (r = 0.307, *p* = 0.006) and Δ12 HbA1c (r = 0.341, *p* = 0.002). Baseline iGlarLixi dose was also positively correlated with Δ3 C-peptide (r = 0.293, *p* = 0.009) and Δ12 C-peptide (r = 0.265, *p* = 0.018). Similarly, lixisenatide dose was positively correlated with Δ3 HbA1c (r = 0.293, *p* = 0.009) and Δ12 HbA1c (r = 0.369, *p* = 0.001). No significant correlations were observed between baseline dose and Δ weight (r = −0.102, *p* = 0.340) or Δ albumin-to-creatinine ratio (r = −0.085, *p* = 0.410). No significant associations were found between baseline dose and changes in lipid parameters (all *p* > 0.05) (Table 8).

Dose-Tertile-Based and adjusted analysis of treatment dose with clinical outcomes are shown in Table 9. Δ3 HbA1c change differed across dose tertiles, with median changes of −0.03% (IQR: 1.95) in the low-dose group, −0.20% (IQR: 1.80) in the medium-dose group, and −0.60% (IQR: 1.45) in the high-dose group (*p* = 0.024; ε^2^ = 0.07). Δ12 HbA1c change showed median changes of −0.20% (IQR: 2.35), −0.40% (IQR: 2.10), and −0.80% (IQR: 1.90) across low-, medium-, and high-dose groups, respectively (*p* = 0.005; ε^2^ = 0.12). Median Δ3 weight change was −1.5 kg (IQR: 1.0), −2.0 kg (IQR: 1.5), and −1.2 kg (IQR: 3.0) (*p* = 0.162; ε^2^ = 0.02), while Δ12 weight change was −3.0 kg (IQR: 2.0), −4.0 kg (IQR: 2.0), and −3.2 kg (IQR: 4.0) across the respective dose groups (*p* = 0.627; ε^2^ < 0.01). Median Δ3 C-peptide change was +0.27 ng/mL (IQR: 1.31), +0.30 ng/mL (IQR: 1.40), and +0.50 ng/mL (IQR: 1.26) (*p* = 0.055; ε^2^ = 0.05), and Δ12 C-peptide change was +0.51 ng/mL (IQR: 1.67), +0.60 ng/mL (IQR: 1.90), and +0.89 ng/mL (IQR: 0.50) (*p* = 0.195; ε^2^ = 0.02) across low-, medium-, and high-dose groups, respectively. Median Δ12 albumin-to-creatinine ratio change was −2.0 mg/g (IQR: 19.0), −2.3 mg/g (IQR: 20.5), and −2.5 mg/g (IQR: 17.0) (*p* = 0.480; ε^2^ < 0.01). In adjusted analyses, baseline treatment dose was associated with Δ3 HbA1c (β = −0.25, 95% CI: −0.45 to −0.05, *p* = 0.013) and Δ12 HbA1c (β = −0.29, 95% CI: −0.51 to −0.07, *p* = 0.010), while no significant associations were observed for Δ3 weight (β = −0.08, 95% CI: −0.25 to 0.09, *p* = 0.340), Δ12 weight (β = 0.11, 95% CI: −0.09 to 0.30, *p* = 0.268), Δ3 C-peptide (β = 0.18, 95% CI: −0.02 to 0.38, *p* = 0.078), Δ12 C-peptide (β = 0.06, 95% CI: −0.16 to 0.29, *p* = 0.576), and Δ12 albumin-to-creatinine ratio (β = 0.13, 95% CI: −0.09 to 0.36, *p* = 0.239) (Table 9).

**Figure 3:**
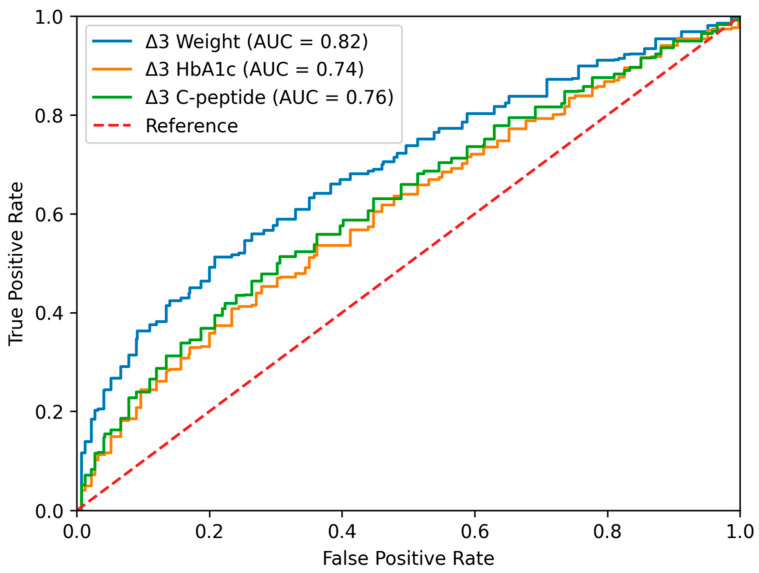
ROC curve analysis of individual early predictors for long-term clinical outcomes.

The results of exploratory ROC analyses evaluating early predictors of long-term clinical outcomes are presented in Table 10. These analyses suggested that early changes in body weight, HbA1c, and C-peptide may be associated with long-term clinical outcomes. ROC curve analysis demonstrated that Δ3 weight predicted ≥3 kg weight loss at 12 months with good discriminative performance (AUC: 0.82, 95% CI: 0.72–0.91), using a cut-off value of −1.5 kg (sensitivity: 78%, specificity: 75%, *p* < 0.001). Δ3 HbA1c predicted ≥1% HbA1c reduction at 12 months with acceptable accuracy (AUC: 0.74, 95% CI: 0.63–0.85; sensitivity: 72%, specificity: 70%, *p* = 0.002). Δ3 C-peptide demonstrated good predictive ability for reduction in albuminuria (AUC: 0.76, 95% CI: 0.65–0.86; sensitivity: 70%, specificity: 73%, *p* < 0.001). Baseline iGlarLixi dose also showed acceptable predictive performance for ≥1% HbA1c reduction (AUC: 0.71, 95% CI: 0.60–0.82, *p* = 0.002) (Table 10, Figure 3).

Exploratory advanced ROC analyses, including DeLong comparisons and Net Reclassification Improvement (NRI) calculations, were performed to evaluate the potential incremental predictive value of combined markers. Given the limited sample size, these findings should be considered hypothesis-generating. The results of these exploratory analyses are presented in Table 11. The AUC for Δ3 weight was 0.82 (95% CI: 0.72–0.91). Δ3 C-peptide had an AUC of 0.76 (95% CI: 0.65–0.86), which was lower than Δ3 weight (ΔAUC = −0.06, *p* = 0.041). Baseline iGlarLixi dose had an AUC of 0.71 (95% CI: 0.60–0.82), also lower than Δ3 weight (ΔAUC = −0.11, *p* = 0.008). The combined model incorporating Δ3 weight and Δ3 C-peptide demonstrated improved discriminative performance (AUC: 0.88, 95% CI: 0.80–0.96), which was significantly higher than Δ3 weight alone (ΔAUC = 0.06, *p* = 0.018).

Reclassification analysis suggested a potential improvement in risk stratification with the combined model compared with individual predictors, with a total NRI of 0.30 (*p* = 0.012) versus Δ3 weight and 0.37 (*p* = 0.008) versus Δ3 C-peptide. However, these findings should be interpreted with caution given the relatively small sample size.

**Figure 4:**
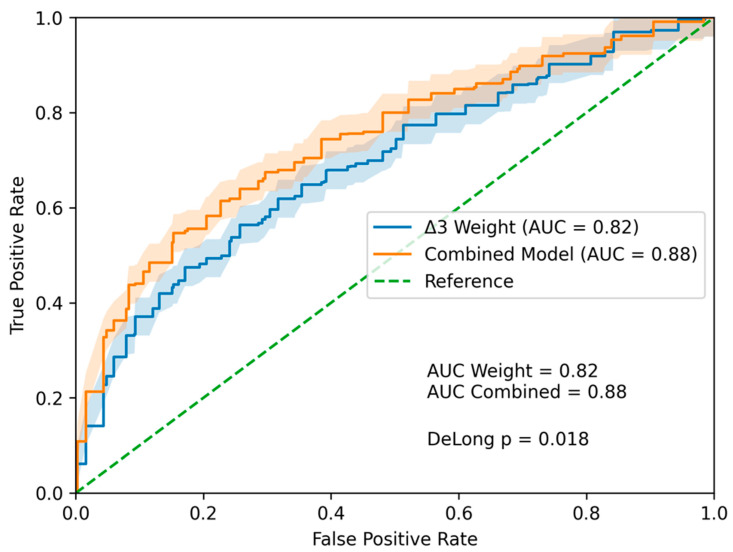
ROC curve analysis of the combined predictive model incorporating Δ3 weight and Δ3 C-peptide.

In predictive performance analysis, Δ3 weight had an AUC of 0.82 (95% CI: 0.72–0.91) with a sensitivity of 78% and specificity of 75% at a cut-off of −1.5 kg. Δ3 C-peptide had an AUC of 0.76 (95% CI: 0.65–0.86) with a sensitivity of 70% and specificity of 73% at a cut-off of +0.20. The combined model demonstrated an AUC of 0.88 (95% CI: 0.80–0.96), with a sensitivity of 84% and specificity of 80% at an optimal probability cut-off of 0.62 (Table 11, Figure 4).

Decision curve analysis suggested that the combined model may provide a net clinical benefit compared with “treat all” and “treat none” strategies across a range of threshold probabilities. However, these findings should be interpreted with caution due to the relatively small sample size and potential risk of overfitting.

## 4. Discussion

In the present study, we evaluated the 12-month real-world effectiveness of iGlarLixi therapy and demonstrated its impact on glycemic control, body weight, endogenous insulin secretion, and renal parameters. Overall, iGlarLixi therapy was associated with a statistically significant, although moderate, reduction in HbA1c levels at 12 months, with clinically meaningful improvement (≥1% reduction) observed in approximately one-quarter of the patients. In parallel, body weight decreased consistently throughout the follow-up period, showing a large effect size. C-peptide levels progressively increased over time, suggesting a potential improvement in endogenous insulin secretion. However, C-peptide measurements alone cannot establish β-cell preservation or restoration, and therefore these findings should be interpreted cautiously. Although median albuminuria values remained relatively stable at the group level, more than half of the patients exhibited individual-level improvement in ACR. Furthermore, early treatment responses demonstrated predictive utility for long-term metabolic and renal outcomes.

Our findings confirm that iGlarLixi provides effective glycemic control in routine clinical practice. The observed HbA1c reduction at 12 months is consistent with real-world studies reporting significant HbA1c improvements following iGlarLixi initiation. Bala et al. demonstrated a significant HbA1c reduction with concurrent weight loss in a large real-world cohort after 24 weeks of treatment [22]. Kis et al. reported consistent reductions in HbA1c across different baseline characteristics, particularly in patients with higher baseline HbA1c levels [12]. However, the magnitude of HbA1c reduction in our study appears more modest compared with randomized controlled trials. Terauchi et al. reported HbA1c reductions of approximately −1.4% in the LixiLan trials [39], whereas the median reduction in our cohort was smaller. This discrepancy likely reflects real-world factors such as heterogeneous patient characteristics, variable adherence, and less intensive titration protocols. Candido et al. also emphasized that real-world outcomes often fall short of clinical trial efficacy due to suboptimal titration and therapeutic inertia [23].

An interesting finding was the significant reduction in HbA1c despite the absence of a statistically significant change in fasting plasma glucose. This discrepancy may be explained by the pharmacologic profile of lixisenatide, which primarily improves postprandial glycemic excursions through delayed gastric emptying and enhanced glucose-dependent insulin secretion. Because HbA1c reflects overall glycemic exposure rather than fasting glucose alone, improvements in postprandial glucose control may contribute substantially to HbA1c reduction.

Importantly, we demonstrated that 26.9% of patients achieved a clinically meaningful HbA1c reduction of ≥1%. This aligns with previous reports showing that a substantial proportion—but not all—patients benefit significantly from fixed-ratio combination therapies, highlighting inter-individual variability in treatment response [19].

Consistent with prior evidence, iGlarLixi therapy was associated with significant and sustained weight reduction. This finding is in line with studies showing that the addition of GLP-1 receptor agonists to basal insulin mitigates insulin-associated weight gain. Bala et al. reported weight reduction alongside HbA1c improvement in real-world settings, while Kis et al. observed weight loss particularly in obese patients [12,22]. Compared with basal insulin intensification alone, fixed-ratio combinations offer a metabolic advantage by targeting both fasting and postprandial glucose while promoting satiety and delaying gastric emptying. Previous randomized trials have also demonstrated weight neutrality or modest weight loss with iGlarLixi compared with weight gain observed with basal insulin therapy [39,40].

One of the notable findings of our study is the progressive increase in C-peptide levels over time, suggesting improved endogenous insulin secretion. This observation is consistent with the glucose-dependent insulinotropic effects of GLP-1 receptor agonists. Novodvorský et al. emphasized that GLP-1-based therapies may reduce β-cell stress and enhance functional responsiveness [36]. While most clinical studies focus primarily on HbA1c outcomes, fewer have evaluated C-peptide dynamics. Our findings contribute to the literature by demonstrating that iGlarLixi may have effects beyond glycemic control, potentially reflecting improved endogenous insulin secretion and reduced glucotoxicity. However, C-peptide measurements alone cannot establish β-cell preservation or restoration, and therefore these findings should be interpreted cautiously. This is supported by mechanistic studies indicating that improved metabolic control may preserve β-cell activity in T2DM [37].

The evaluation of renal parameters revealed a nuanced pattern. Although ACR showed statistically significant paired changes over time, median values remained largely unchanged. This apparent discrepancy reflects the heterogeneity of individual responses, as more than half of patients exhibited a reduction in albuminuria. This finding is consistent with the growing body of evidence suggesting that GLP-1 receptor agonists may exert renoprotective effects. Meier et al. highlighted that GLP-1 RAs may reduce albuminuria through multiple mechanisms, including improved glycemic control, weight reduction, and anti-inflammatory effects [26]. Muskiet et al. also demonstrated favorable renal effects of lixisenatide in patients with T2DM [30]. However, the modest group-level effect observed in our study suggests that renal benefits may be more variable and potentially dependent on baseline renal status and duration of diabetes. Therefore, our results support the concept that albuminuria reduction occurs at the individual level rather than uniformly across all patients.

A key strength of this study is the evaluation of early predictors of long-term outcomes. We demonstrated that early changes in body weight, HbA1c, and C-peptide were associated with 12-month outcomes, with acceptable to good predictive performance in ROC analysis. This is clinically relevant, as early response markers may help guide treatment optimization. The combined model incorporating Δ3 weight and Δ3 C-peptide showed improved discriminative ability compared with individual predictors. While reclassification analysis (NRI) suggested incremental predictive value, these findings should be interpreted cautiously due to the relatively small sample size. Similar concerns regarding overestimation of predictive improvement in small cohorts have been highlighted in methodological studies. From a clinical perspective, early identification of responders and non-responders may facilitate personalized treatment strategies, particularly in heterogeneous T2DM populations. However, the predictive analyses should be interpreted with caution because they were performed in a relatively small cohort without external validation. Therefore, the reported ROC, NRI, and DCA findings should be viewed as exploratory and hypothesis-generating rather than evidence of established clinical prediction models.

Interestingly, baseline treatment dose was positively correlated with ΔHbA1c values, indicating that higher baseline doses were associated with less favorable glycemic change. This finding contrasts with a simple dose–response expectation and may reflect confounding by indication, where patients requiring higher initial doses may have more advanced disease or greater insulin resistance. Previous real-world studies have also emphasized that treatment effectiveness depends not only on dose but also on titration adequacy and patient-specific factors [19,23]. Therefore, our findings highlight the importance of individualized dose adjustment rather than relying solely on baseline dosing. Furthermore, correlation analyses and dose-tertile comparisons reflect different statistical approaches and therefore should not be interpreted as directly equivalent measures of dose–response. The correlation analyses evaluated continuous dose–response relationships at the individual level, whereas the tertile analyses compared predefined dose categories. In addition, patients receiving higher baseline doses may have had more advanced disease, greater insulin resistance, or poorer baseline glycemic control, potentially introducing confounding by indication. These factors may have contributed to apparent differences between individual-level and group-level analyses. Moreover, treatment response may not be strictly linear across the dose spectrum, and nonlinear effects cannot be excluded in a real-world cohort of this size.

This study has several strengths. First, it provides long-term (12-month) real-world data on iGlarLixi, extending beyond the shorter follow-up periods commonly reported in the literature. Second, it integrates multiple clinically relevant parameters, including glycemic control, body weight, renal markers, and C-peptide. Third, the analysis of early predictors and composite modeling adds practical clinical value.

However, some limitations should be acknowledged. The retrospective single-center design may limit generalizability. In addition, the absence of a control group limits causal inference. Without comparison to basal insulin alone or another treatment intensification strategy, it is not possible to determine whether the observed changes were specifically attributable to iGlarLixi. The sample size, although adequate for primary outcomes, may limit the robustness of advanced predictive analyses such as NRI and DCA. Furthermore, information regarding treatment adherence, dietary compliance, physical activity, and detailed insulin titration practices was not systematically available because of the retrospective design. These factors may have influenced treatment outcomes and should be considered when interpreting the findings. In addition, concomitant medications, particularly SGLT2 inhibitors, ACE inhibitors/ARBs, and statins, may have influenced metabolic and renal outcomes. Although these therapies reflect routine clinical practice, residual confounding cannot be completely excluded. In addition, changes in concomitant medications during follow-up were not systematically available and therefore could not be incorporated into the analyses. Furthermore, hypoglycemia and other safety outcomes were not systematically recorded and therefore could not be evaluated. Given that reduced hypoglycemia risk represents a potential advantage of fixed-ratio insulin/GLP-1 receptor agonist combinations, the absence of safety data should be considered when interpreting treatment effectiveness. Furthermore, the predictive analyses were conducted in a relatively small sample and without an independent validation cohort. Consequently, the reported discrimination and reclassification metrics may be susceptible to optimism bias and overfitting. Finally, missing data for certain parameters, particularly renal markers, may introduce bias.

## 5. Conclusions

In conclusion, iGlarLixi therapy was associated with significant improvements in glycemic control and body weight over a 12-month period in a real-world setting. Clinically meaningful HbA1c reduction was achieved in a subset of patients, highlighting variability in treatment response. While group-level renal parameters remained largely stable, individual-level reductions in albuminuria were observed in a substantial proportion of patients. Early changes in body weight, HbA1c, and C-peptide demonstrated potential predictive value for long-term outcomes, supporting their use as practical markers for treatment monitoring. These findings suggest that iGlarLixi represents an effective and clinically useful option for treatment intensification in patients with type 2 diabetes mellitus.

## Figures and Tables

**Table 1:** Baseline demographic and clinical characteristics of the study population (N = 78).

Variable	Value (N = 78) Median (Interquartile Range, IQR)
Age (years)	58 (10)
Female, n (%)	61 (78.2%)
Male, n (%)	17 (21.8%)
Duration of diabetes (years)	10 (8)
Body weight (kg)	93.0 (18.25)
BMI (kg/m^2^)	35.65 (7.32)
Fasting plasma glucose (mg/dL)	182.0 (113.0)
HbA1c (%)	9.0 (2.43)
Creatinine (mg/dL)	0.725 (0.32)
eGFR (mL/min/1.73 m^2^)	95.0 (32.0)
Uric acid (mg/dL)	4.61 (2.01)
Albumin/creatinine ratio (mg/g) (ACR)	15.30 (48.61)
Protein/creatinine ratio (mg/g) (PCR)	88.5 (82.48)
Total cholesterol (mg/dL)	209 (52)
LDL-C (mg/dL)	113.5 (47.5)
HDL-C (mg/dL)	52 (15)
Triglycerides (mg/dL)	201 (117)
C-peptide (ng/mL)	2.46 (2.59)

All continuous variables are presented as median (interquartile range, IQR). Categorical variables are presented as number (percentage). BMI: body mass index; eGFR: estimated glomerular filtration rate.

**Table 2:** Baseline comorbidities and concomitant medications.

Variable	n (%)
Hypertension	48 (61.5)
Dyslipidemia	52 (66.7)
Obesity (BMI ≥ 30 kg/m^2^)	61 (78.2)
Coronary artery disease	14 (17.9)
Chronic kidney disease	9 (11.5)
Hepatic steatosis	29 (37.2)
Normoalbuminuria (<30 mg/g)	45 (57.7)
Microalbuminuria (30–300 mg/g)	26 (33.3)
Macroalbuminuria (>300 mg/g)	7 (9.0)
Metformin	67 (85.9)
SGLT2 inhibitor	38 (48.7)
DPP-4 inhibitor	24 (30.8)
Sulfonylurea	19 (24.4)
Basal insulin	43 (55.1)
ACE inhibitor/ARB	45 (57.7)
Statin therapy	49 (62.8)

**Table 3:** Changes in anthropometric and glycemic parameters over time.

Variable	Baseline Median (IQR)	3 Months Median (IQR)	12 Months Median (IQR)	*p*-Value (0–3 mo)	*p*-Value (0–12 mo)	*p*-Value (3–12 mo)	Effect Size (r)
Body weight (kg)	93.0 (18.25)	91.0 (17.0)	89.5 (17.0)	<0.001	<0.001	<0.001	0.52
BMI (kg/m^2^)	35.65 (7.32)	35.41 (7.18)	34.74 (7.11)	<0.001	<0.001	<0.001	0.50
Fasting glucose (mg/dL)	182.0 (113.0)	168.0 (95.0)	165.0 (100.0)	0.612	0.085	0.210	0.18
HbA1c (%)	9.0 (2.43)	9.0 (1.95)	8.2 (2.33)	0.521	0.015	0.006	0.34

Values are presented as median (interquartile range, IQR). *p*-values were calculated using the Friedman test for overall comparison and Wilcoxon signed-rank test for pairwise comparisons. Effect size (r) was calculated as Z/√N and interpreted as small (0.1–0.3), moderate (0.3–0.5), and large (>0.5).

**Table 4:** Changes in renal function parameters over time.

Variable	Baseline Median (IQR)	3 Months Median (IQR)	12 Months Median (IQR)	*p*-Value (0–3 mo)	*p*-Value (0–12 mo)	*p*-Value (3–12 mo)	Effect Size (r)
Creatinine (mg/dL)	0.725 (0.32)	0.720 (0.24)	0.720 (0.29)	0.022	0.776	0.086	0.12
eGFR (mL/min/1.73 m^2^)	95.0 (32.0)	96.0 (32.0)	97.63 (27.78)	0.064	0.703	0.290	0.10
Albumin/Creatinine ratio (mg/g)	15.30 (48.61)	19.57 (38.22)	15.34 (33.51)	0.194	0.012	0.006	0.28
Protein/Creatinine ratio (mg/g)	88.5 (82.48)	71.35 (76.58)	68.30 (62.33)	0.005	0.002	0.020	0.32

Values are presented as median (interquartile range, IQR). *p*-values were calculated using the Friedman test for overall comparison and Wilcoxon signed-rank test for pairwise comparisons. Effect size (r) was calculated as Z/√N. Changes in ACR represent paired individual-level differences and may not correspond to differences in group medians.

**Table 5:** Changes in lipid profile and biochemical parameters.

Variable	Baseline Median (IQR)	3 Months Median (IQR)	12 Months Median (IQR)	*p*-Value (0–3 mo)	*p*-Value (0–12 mo)	*p*-Value (3–12 mo)	Effect Size (r)
Total cholesterol (mg/dL)	209 (52)	202 (58)	194 (43.5)	0.764	0.002	0.022	0.30
LDL cholesterol (mg/dL)	113.5 (47.5)	111 (29)	102.86 (45)	0.748	0.096	0.016	0.26
HDL cholesterol (mg/dL)	52 (15)	52 (15)	50 (15.6)	0.512	0.399	0.283	0.10
Triglycerides (mg/dL)	201 (117)	193 (120.9)	196 (112.5)	0.876	0.381	0.686	0.08
C-peptide (ng/mL)	2.46 (2.59)	2.66 (2.53)	3.00 (2.93)	0.030	0.016	0.001	0.33
TSH (mIU/L)	1.96 (1.39)	1.69 (1.18)	1.76 (1.04)	0.081	0.009	0.264	0.24
Uric acid (mg/dL)	4.61 (2.01)	4.71 (1.72)	4.75 (1.89)	0.386	0.289	0.280	0.09

Values are presented as median (interquartile range, IQR). *p*-values were calculated using the Friedman test for overall comparison and Wilcoxon signed-rank test for pairwise comparisons. Effect size (r) was calculated as Z/√N and interpreted as small (0.1–0.3), moderate (0.3–0.5), and large (>0.5).

**Table 6:** Median changes (Δ3 and Δ12) in key clinical parameters.

Variable	Δ3 Median (IQR)	Δ12 Median (IQR)	*p*-Value	Effect Size (r)
Body weight (kg)	−1.88 (1.00)	−3.13 (2.55)	<0.001	0.52
HbA1c (%)	−0.03 (1.95)	−0.20 (2.35)	0.015	0.34
C-peptide (ng/mL)	+0.27 (1.31)	+0.51 (1.67)	0.016	0.33
Albumin/Creatinine ratio (mg/g)	−1.85 (18.60)	−2.56 (19.85)	0.012	0.28

Δ3 represents the change from baseline to 3 months, and Δ12 represents the change from baseline to 12 months. Values are presented as median (interquartile range, IQR). *p*-values were calculated using the Wilcoxon signed-rank test. Effect size (r) was calculated as Z/√N. Because Δ values were calculated from paired individual patient data, median Δ values do not necessarily equal the arithmetic difference between group-level median values at baseline and follow-up.

**Table 7:** Correlation analysis between Δ3 and Δ12 changes.

Variable Pair	Correlation Coefficient (r)	*p*-Value
Δ3 weight vs. Δ12 weight	0.801	<0.001
Δ3 HbA1c vs. Δ12 HbA1c	0.639	<0.001
Δ3 C-peptide vs. Δ12 C-peptide	0.658	<0.001
Δ3 weight vs. Δ12 HbA1c	−0.268	0.018
Δ3 weight vs. Δ3 C-peptide	−0.370	0.001
Δ12 weight vs. Δ12 C-peptide	−0.262	0.021
Δ12 C-peptide vs. Δ3 ACR	−0.309	0.006
Δ12 C-peptide vs. Δ12 ACR	−0.243	0.035
Δ3 HbA1c vs. Δ3 weight	−0.092	0.425
Δ12 HbA1c vs. Δ12 weight	−0.221	0.054

**Table 8:** Associations between treatment dose and clinical outcomes.

Variable Pair	Correlation Coefficient (r)	*p*-Value
Baseline iGlarLixi dose vs. Δ3 HbA1c	0.307	0.006
Baseline iGlarLixi dose vs. Δ12 HbA1c	0.341	0.002
Baseline iGlarLixi dose vs. Δ3 C-peptide	0.293	0.009
Baseline iGlarLixi dose vs. Δ12 C-peptide	0.265	0.018
Lixisenatide dose vs. Δ3 HbA1c	0.293	0.009
Lixisenatide dose vs. Δ12 HbA1c	0.369	0.001
Baseline dose vs. Δ weight	−0.102	0.340
Baseline dose vs. Δ ACR	−0.085	0.410
Baseline dose vs. Δ lipid parameters	—	All *p* > 0.05

Associations were evaluated using Spearman’s rank correlation coefficient. Δ3 and Δ12 represent changes from baseline to 3 months and 12 months, respectively. Lipid parameters include total cholesterol, LDL-C, HDL-C, and triglycerides. No significant correlations were observed between treatment dose and changes in lipid parameters.

**Table 9:** Dose-Tertile-Based and adjusted analysis of treatment dose with clinical outcomes.

Outcome	Low Dose n = 29 Median (IQR)	Medium Dose n = 32 Median (IQR)	High Dose n = 17 Median (IQR)	*p* (KW)	Effect Size ε^2^	Adjusted Β for Dose *	95% CI	*p* (Adj)
Δ3 HbA1c change (%)	−0.03 (1.95)	−0.20 (1.80)	−0.60 (1.45)	0.024	0.07	−0.25	−0.45 to −0.05	0.013
Δ12 HbA1c change (%)	−0.20 (2.35)	−0.40 (2.10)	−0.80 (1.90)	0.005	0.12	−0.29	−0.51 to −0.07	0.010
Δ3 weight change (kg)	−1.5 (1.0)	−2.0 (1.5)	−1.2 (3.0)	0.162	0.02	−0.08	−0.25 to 0.09	0.340
Δ12 weight change (kg)	−3.0 (2.0)	−4.0 (2.0)	−3.2 (4.0)	0.627	<0.01	0.11	−0.09 to 0.30	0.268
Δ3 C-peptide change (ng/mL)	+0.27 (1.31)	+0.30 (1.40)	+0.50 (1.26)	0.055	0.05	0.18	−0.02 to 0.38	0.078
Δ12 C-peptide change (ng/mL)	+0.51 (1.67)	+0.60 (1.90)	+0.89 (0.50)	0.195	0.02	0.06	−0.16 to 0.29	0.576
Δ12 ACR change (mg/g)	−2.0 (19.0)	−2.3 (20.5)	−2.5 (17.0)	0.480	<0.01	0.13	−0.09 to 0.36	0.239

Values are presented as median (interquartile range, IQR). Dose tertiles were defined according to baseline insulin glargine/lixisenatide dose: low dose, 16–24 U; medium dose, 26–32 U; high dose, 34–60 U. Between-group comparisons were performed using the Kruskal–Wallis (KW) test. Effect size was calculated using epsilon-squared (ε^2^). Δ values were calculated as follow-up minus baseline; therefore, negative values indicate reductions, whereas positive values indicate increases. * Adjusted (Adj) β values are standardized coefficients derived from linear regression models adjusted for baseline BMI.

**Table 10:** ROC curve analysis of early predictors for long-term clinical outcomes.

Predictor	Outcome	AUC (95% CI)	Cut-Off	Sensitivity (%)	Specificity (%)	*p*-Value
Δ3 weight (kg)	≥3 kg weight loss at 12 months	0.82 (0.72–0.91)	−1.5 kg	78	75	<0.001
Δ3 HbA1c (%)	≥1% HbA1c reduction at 12 months	0.74 (0.63–0.85)	−0.5%	72	70	0.002
Δ3 C-peptide (ng/mL)	Reduction in albuminuria	0.76 (0.65–0.86)	+0.20	70	73	<0.001
Baseline iGlarLixi dose	≥1% HbA1c reduction at 12 months	0.71 (0.60–0.82)	30 units	68	69	0.002

ROC: receiver operating characteristic; AUC: area under the curve; CI: confidence interval. Cut-off values were determined using the Youden index. Sensitivity and specificity correspond to optimal cut-off thresholds. AUC values were interpreted as acceptable (0.70–0.80), good (0.80–0.90), and excellent (>0.90).

**Table 11:** Advanced ROC Analysis, DeLong Comparison, Net Reclassification Improvement (NRI), and Combined Model Performance.

**Model/Predictor**	**AUC (95% CI)**	**Reference Model**	**ΔAUC**	**DeLong *p*-Value**
Δ3 weight	0.82 (0.72–0.91)	—	—	—
Δ3 C-peptide	0.76 (0.65–0.86)	Δ3 weight	−0.06	0.041
Baseline dose	0.71 (0.60–0.82)	Δ3 weight	−0.11	0.008
Combined model (Δ3 weight + C-peptide)	0.88 (0.80–0.96)	Δ3 weight	+0.06	0.018
**Comparison**	**Event NRI**	**Non-Event NRI**	**Total NRI**	***p*-Value**
Combined model vs. Δ3 weight	0.18	0.12	0.30	0.012
Combined model vs. Δ3 C-peptide	0.22	0.15	0.37	0.008
**Predictive Performance of Combined Model**	**AUC (95% CI)**	**Sensitivity (%)**	**Specificity (%)**	**Optimal Cut-Off**
Δ3 weight	0.82 (0.72–0.91)	78	75	−1.5 kg
Δ3 C-peptide	0.76 (0.65–0.86)	70	73	+0.20
Combined model	0.88 (0.80–0.96)	84	80	Probability = 0.62

DeLong test was used to compare AUC values between models. Net Reclassification Improvement (NRI) was calculated to assess the incremental predictive value of the combined model. The combined model was constructed using logistic regression including Δ3 weight and Δ3 C-peptide as predictors. Optimal cut-off values were determined using the Youden index.

## Data Availability

Data is contained within the article.

## Abbreviations

ACR	Albumin-to-Creatinine Ratio
ADA	American Diabetes Association
ALP	Alkaline Phosphatase
ALT	Alanine Aminotransferase
AST	Aspartate Aminotransferase
AUC	Area Under the Curve
BMI	Body Mass Index
CI	Confidence Interval
C-peptide	Connecting Peptide
DCA	Decision Curve Analysis
eGFR	Estimated Glomerular Filtration Rate
GLP-1 RA	Glucagon-Like Peptide-1 Receptor Agonist
GGT	Gamma-Glutamyl Transferase
HbA1c	Glycated Hemoglobin
HDL-C	High-Density Lipoprotein Cholesterol
iGlarLixi	Insulin Glargine/Lixisenatide
IQR	Interquartile Range
LDL-C	Low-Density Lipoprotein Cholesterol
NRI	Net Reclassification Improvement
PCR	Protein-to-Creatinine Ratio
ROC	Receiver Operating Characteristic
SPSS	Statistical Package for the Social Sciences
T2DM	Type 2 Diabetes Mellitus
TSH	Thyroid-Stimulating Hormone
